# Modeling and Simulations of 4H-SiC/6H-SiC/4H-SiC Single Quantum-Well Light Emitting Diode Using Diffusion Bonding Technique

**DOI:** 10.3390/mi12121499

**Published:** 2021-11-30

**Authors:** Muhammad Haroon Rashid, Ants Koel, Toomas Rang, Nadeem Nasir, Haris Mehmood, Salman Cheema

**Affiliations:** 1 Department of Textile Engineering, National Textile University, Faisalabad 37610, Pakistan; 2 Thomas Johann Seebeck Department of Electronics, Tallinn University of Technology, Ehitajate tee 5, 12616 Tallinn, Estonia; ants.koel@taltech.ee (A.K.); toomas.rang@taltech.ee (T.R.); 3 Department of Applied Sciences, National Textile University, Faisalabad 37610, Pakistan; nadeemnasir@ntu.edu.pk (N.N.); saqvn.cheema@gmail.com (S.C.); 4 Department of Electrical Engineering, Information Technology University (ITU) of the Punjab, Lahore 54600, Pakistan; haris.mehmood@itu.edu.pk

**Keywords:** 4H-SiC, 6H-SiC, light-emitting diode, diffusion bonding, diffusion welding, quantum well, silicon carbide, edge-emitting led

## Abstract

In the last decade, silicon carbide (SiC) has emerged as a potential material for high-frequency electronics and optoelectronics applications that may require elevated temperature processing. SiC exists in more than 200 different crystallographic forms, referred to as polytypes. Based on their remarkable physical and electrical characteristics, such as better thermal and electrical conductivities, 3C-SiC, 4H-SiC, and 6H-SiC are considered as the most distinguished polytypes of SiC. In this article, physical device simulation of a light-emitting diode (LED) based on the unique structural configuration of 4H-SiC and 6H-SiC layers has been performed which corresponds to a novel material joining technique, called diffusion welding/bonding. The proposed single quantum well (SQW) edge-emitting SiC-based LED has been simulated using a commercially available semiconductor device simulator, SILVACO TCAD. Moreover, by varying different design parameters, the current-voltage characteristics, luminous power, and power spectral density have been calculated. Our proposed LED device exhibited promising results in terms of luminous power efficiency and external quantum efficiency (EQE). The device numerically achieved a luminous efficiency of 25% and EQE of 16.43%, which is at par performance for a SQW LED. The resultant LED structure can be customized by choosing appropriate materials of varying bandgaps to extract the light emission spectrum in the desired wavelength range. It is anticipated that the physical fabrication of our proposed LED by direct bonding of SiC-SiC wafers will pave the way for the future development of efficient and cost-effective SiC-based LEDs.

## 1. Introduction

The phenomenon of electroluminescence was observed for the very first time in the crystal of silicon carbide (SiC) in 1907. At that time of earlier development stages, the light emission process through semiconductors was not well understood [1]. However, a significant progress in the field of solid-state light-emitting diodes (LEDs) had been made in the 1990s with the development of the first blue LED [2]. In recent years, the astonishing features of solid-state LEDs such as high luminous efficiency, high color rendering index (CRI), and longevity have made them promising candidates to replace conventional light sources [3]. The current research trends involve a systematic investigation of a variety of organic/inorganic materials for a wide range of optoelectronic applications [4,5,6,7,8,9,10].

SiC is a wide bandgap compound semiconductor material that is highly suitable for optoelectronic applications involving operations at higher temperatures. It possesses high thermal stability, enhanced electric field maintenance, and a remarkable physical strength compared to that of Silicon (Si). It is also highly suitable for light-emitting applications. SiC exists in several polytypic forms among which 4H-SiC and 6H-SiC have gained attention due to their attractive physical and electronics attributes [11]. In recent years, SiC has attained significant importance for many quantum scale optoelectronic applications, especially for single-photon emitter (SPE) applications [12]. Nuclear spins of SiC can exist in spin-free states [13] that allow color coherence and long coherence times in SiC quantum devices [14,15]. In SiC-based quantum optoelectronic devices, charge injection results in an increased background emission of light [16]. But although the generation of single photons has been reported in several systems, none of them is suitable for room temperature applications (especially in telecommunication services). The development of SiC LEDs with light emission in the visible and near-infrared regions have also been reported in the literature [17]. Doped SiC can give high quantum efficiencies to provide high donor-to-acceptor luminescence [18].

Moreover, SiC is an extraordinary candidate for high-power optoelectronic applications. Its light emission characteristics have several attractive attributes such as higher efficiency, higher electrical conductivity, longer lifetime, and higher output electrical power compared to that of the conventional LEDs. In conventional LEDs, for high-power applications, heat dissipation becomes a crucial challenge that must be overcome. There are many other factors such as current crowding, Auger recombination, electron leakage, and the polarization effect that degrade the performance of the device in terms of efficiency [19]. Overheating of LEDs can be avoided by using SiC due to its extremely low thermal expansion and high thermal conductivity. The light emission and extraction from SiC-based LEDs have not been discussed in detail in the literature since it is extremely challenging to understand its optoelectronics attributes [20,21]. The focus of recent research has been to improve the performance of the LEDs with different fabrication techniques [22,23]. In order to extract the maximum performance from the devices, the majority of the modern solid-state devices have been designed and developed using heterostructure configuration. The development of various optoelectronic devices (i.e., LEDs and lasers) is based on the realization of the double heterostructure configuration. The benefits of utilizing a double-heterostructure scheme in LED include optical confinement as well as minimizing the reabsorption probability of emitted photons [24]. 

The motivation of our research is to contribute to the theoretical research by investigating the fabrication feasibility of SiC-based LEDs using a novel technique; called diffusion bonding/welding. For this purpose, a SiC-based edge-emitting LED has been simulated in this work via a novel technique of diffusion welding. The edge-emitting LEDs have been primarily used as a light source in fiber optic communication links. The diffusion welding or diffusion bonding method is used to join dissimilar materials (usually for making different metal-metal and metal-semiconductor interfaces). The working principle is based on the solid-state diffusion process in which atoms diffuse from a higher concentration to a lower concentration. This method has several economic advantages over state-of-the-art epitaxial techniques. This process reduces the fabrication complexity, processing time, and allows high material utilization [25]. This method has been rarely used to make semiconductor-semiconductor interfaces. The state-of-the-art epitaxial growth techniques for fabricating a SiC-SiC heterostructure are complex and require higher deposition temperatures [26]. The device fabrication from the conventional epitaxial growth techniques results in the wastage of semiconductor materials due to evaporation of the materials [27,28,29]. 

For the purpose of analysis and attaining the fabrication feasibility, the prototype device’s performance parameters are first optimized by employing simulators before the commencement of the physical fabrication process. In this work, the fabrication feasibility of our proposed LED structure has been investigated using a commercially available semiconductor device simulator (SILVACO TCAD) via the diffusion welding approach. This technique has been successfully used to fabricate metallic contacts for SiC-based power electronics devices. However, it has rarely been used for the fabrication of SiC-SiC based heterostructures due to the extreme hardness of SiC wafers and their ability to withstand higher temperatures. Physical fabrication of SiC-SiC heterostructures using our proposed technique could be a breakthrough in semiconductor processing technology [30]. Our research group at Tallinn University of Technology has been working to fabricate SiC-SiC heterostructures by direct bonding of wafers for the last few years. Jana et al. proposed and fabricated SiC diode-based voltage doubler by using the diffusion welding principle. They implemented aluminum foil as a connecting material to join the diodes [31]. A similar concept has been reported by Natalja et al. to join SiC-based diodes [32]. Oleg et al. successfully fabricated a prototype of SiC-based Schottky diodes stack by the diffusion welding process [33].

It is pertinent to mention that the fabrication of our proposed/simulated device may need wafer surface treatment so that the surface may appear smooth and homogenous. The optimized temperature and pressure required to bond the wafer successfully need to be investigated for prospective physical fabrication of our proposed device. As far as the epitaxial growth of 6H-SiC is concerned, it has been reported in the literature. Epi-layer growth of 6H-SiC on 6H-SiC substrate has been performed via chemical vapor deposition in the past [34]. Satoshi Tamura et al. successfully deposited high purity 6H-SiC by cold chemical vapor deposition [35]. Tsunenobu et al. also reported epitaxial growth of 6H-SiC in a step-controlled epitaxy [36]. Deposition of SiC epitaxial layers with chemical vapor deposition has also been reported in the literature [37]. Surface treatment of thick wafers of 4H-SiC is required to get a smooth surface for better physical bonding of SiC-SiC wafers using diffusion bonding/welding techniques. Chemical etching of 4H-SiC in the presence of Platinum catalyst could be a promising technique for polishing and flattening SiC wafer to get better adhesion for the diffusion welding process [38]. Catalytic etching is a damage-free technique for the polishing of 4H-SiC wafers [39].

In this article, a novel design of SiC-SiC based double hetero-structure edge-emitting LED has been proposed using the diffusion welding process. In the SILVACO TCAD simulator, a thin active layer of intrinsic 6H-SiC has been sandwiched between heavily doped P-type and N-type 4H-SiC to make a single quantum-well (SQW) double hetero-structure 4H-SiC/6H-SiC/4H-SiC LED. To the best of our knowledge, this configuration of SiC LED with our proposed technique has not been reported yet in the literature. Physical fabrication of the proposed LED structure with diffusion welding/bonding will simplify the fabrication process and reduce the device fabrication cost (as the diffusion welding process is simple with a low running cost).

## 2. Materials and Methods

**Figure 1:**
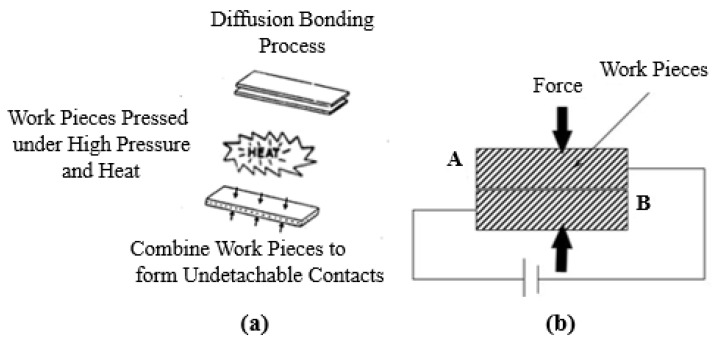
Schematic of diffusion welding process (**a**) Bonding of two wafers under high pressure and temperature; (**b**) Direct bonding of wafers A and B under high pressure [25].

**Figure 2:**
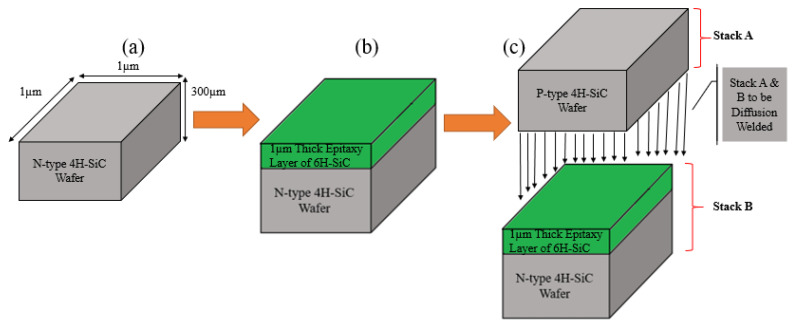
Simulation steps of proposed SiC-based light-emitting diode (**a**) 300 µm thick wafer of N-type 4H-SiC; (**b**) deposition of an epitaxial layer of intrinsic 6H-SiC on 300 µm thick wafer of N-type 4H-SiC; (**c**) diffusion welding of 300 µm thick p-type 4H-SiC (Stack A) wafer with Stack B (n-type 4H-SiC and 6H-SiC).

This section elucidates the methodology that has been adopted for the simulations of the proposed device. In the diffusion welding technique, dissimilar thick material sheets are pressed against each other under high pressure and temperature to make their junctions. The schematic of the process has been shown in Figure 1. It has been shown in this figure that a thick sheet of material-A is being joined with material-B under high pressure and temperature. The same concept has been used for the microscale simulations of our proposed SiC-based SQW LED with a SILVACO TCAD. For our simulated device, the diffusion welding technique has been realized by joining the thick wafers of SiC polytypes directly with each other instead of depositing epitaxial layers. Different simulation steps have been shown in Figure 2. A 300 µm thick wafer of N-type 4H-SiC with a surface area of 1 × 1 µm^2^ has been defined in SILVACO TCAD (as shown in Figure 2a). In the next step, 1 µm thin epi-layer of intrinsic 6H-SiC has been deposited on N-type 4H-SiC substrate to form a stack, as shown in Figure 2b. In the final step, stack B (N-type 4H-SiC and intrinsic epi-layer of 6H-SiC) has been joined with a 300 µm thick wafer of P-type 4H-SiC (Stack A) same as conducted in the diffusion bonding approach (which is illustrated in Figure 2c). 4H-SiC wafers are commercially available with a thickness of 300 µm. Keeping the prospective physical fabrication and availability of the materials in mind, the same thickness (i.e., 300 µm) has been chosen for simulations.

**Figure 3:**
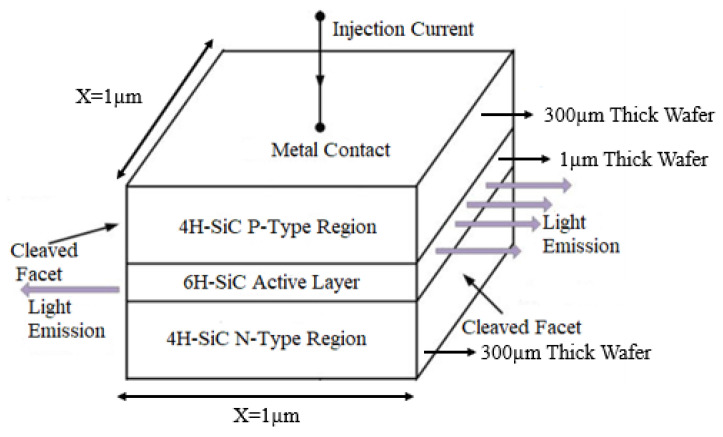
Schematic of 4H-SiC/6H-SiC/4H-SiC single quantum-well double hetero-structure edge-emitting LED.

**Figure 4:**
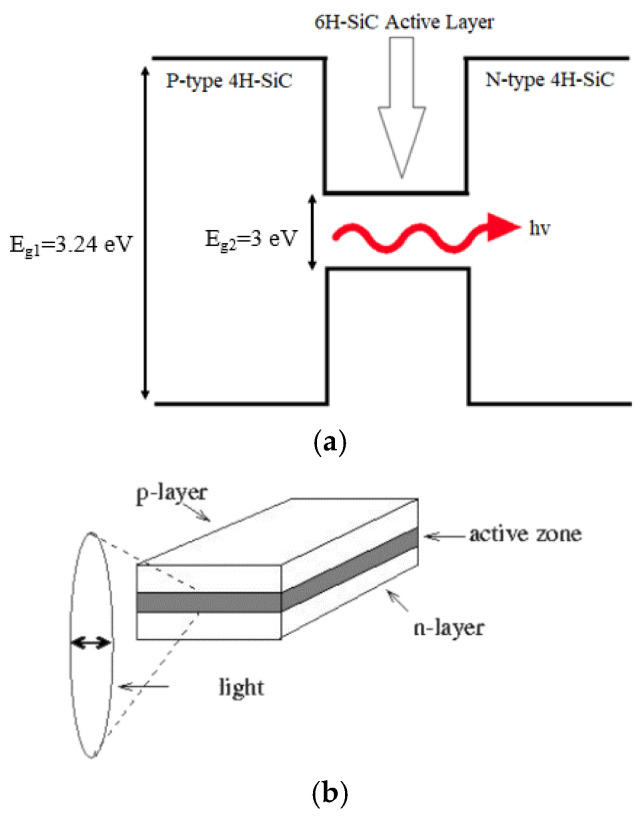
(**a**) Schematic of band profile of 4H-SiC/6H-SiC/4H-SiC LED where E_g1_ is bandgap of 4H-SiC and E_g2_ is bandgap of 6H-SiC; (**b**) emission of light from active layer region of edge-emitting LED.

Heavily doped N- and P-type 4H-SiC layers with concentrations of 10^18^ cm^−3^ have been used in the simulated LED. The final simulated structure based on the above-mentioned steps has been depicted in Figure 3. This type of LED is called edge-emitting LED as light is being emitted from the edges (not from the surface), as shown in Figure 3. Edge emitting LEDs are used in optical communication links as a source of light [40]. As the bandgap of 4H-SiC is larger than that of 6H-SiC, therefore an active layer of intrinsic 6H-SiC forms a quantum-well between 4H-SiC wafers (as illustrated in Figure 4a). An emitted photon from the quantum-well is shown with a red color wave of light in Figure 4a.

Furthermore, we considered a direct bonding process for our simulations since the diffusion welding process is also applicable for extremely thin sheets of materials [25]. Our proposed SiC-based SQW-LED can also be fabricated by the integration of epitaxial growth (a state-of-the-art technique used to fabricate SiC device) [41] and diffusion bonding technique (solid-state material joining technique) [25,42]. Several appropriate physical models related to LEDs have been used during the simulations. Polarization modeling is critical for Wurtzite (WZ) materials as the effect of polarization in WZ materials can cause quantum confinement in QW LEDs. This phenomenon plays an important role in the reduction of radiative recombination in LEDs [43]. The polarization model has been implemented and enabled by the POLARIZATION command in MODEL statement in SILVACO TCAD.

Additionally, an analytical low field mobility model that is dependent on temperature and concentration of charge carriers has also been implemented. In this model, electron and hole mobilities are first defined by MUNO and MUPO parameters. Then, this model is enabled by the ANALYTIC statement under MODELS. This model relates the low-field carrier’s mobility to the concentration of impurities and temperature. This model was proposed by Caughey and Thomas [44,45]. The specific material’s parameters for this model related to 4H-SiC and 6H-SiC are given by Lades [46]. The bandgap narrowing model is an important parameter for heavily doped devices. As N- and P-type regions of the proposed LED are heavily doped, this model plays a crucial role in assessing electrical performance. This model has been enabled by using the BGN statement. Shockely-Read-Hall model is used to fix minority charge carrier’s lifetime and it is enabled by SRH statement in SILVACO TCAD. Similarly, the Auger (AUGER) model is used for the direct transition of carriers. Selberherr’s model has been used for temperature-dependent parameters and it is enabled by the IMPACT SELB statement. Several material’s related parameters such as bandgap, permittivity, electron affinity, the electron density of states, holes density of states, charge carrier’s lifetime, and mobility of charge carriers have been used during the implementation of different physical models. Most of the materials-related parameters have been taken from Atlas user’s manual [47] and are written in Table 1.

## 3. Results

In this section, the results obtained from simulated LED have been included and discussed in detail. Several results demonstrating the performance of the simulated device like energy band profile, current-voltage (IV) characteristics, luminous power, and power spectral density as a function of wavelength have been presented. The plots are generated through SILVACO TCAD Software using TONYPLOT (graphical module of TCAD to visualize results of simulated devices). TONYPLOT is a graphical user interface that is used with all SILVACO simulators to generate graphs. A comprehensive detail about this graphical user interface can be found at this reference [48].

### 3.1. Energy Band Profile and IV-Characteristics of Simulated LED

**Figure 5:**
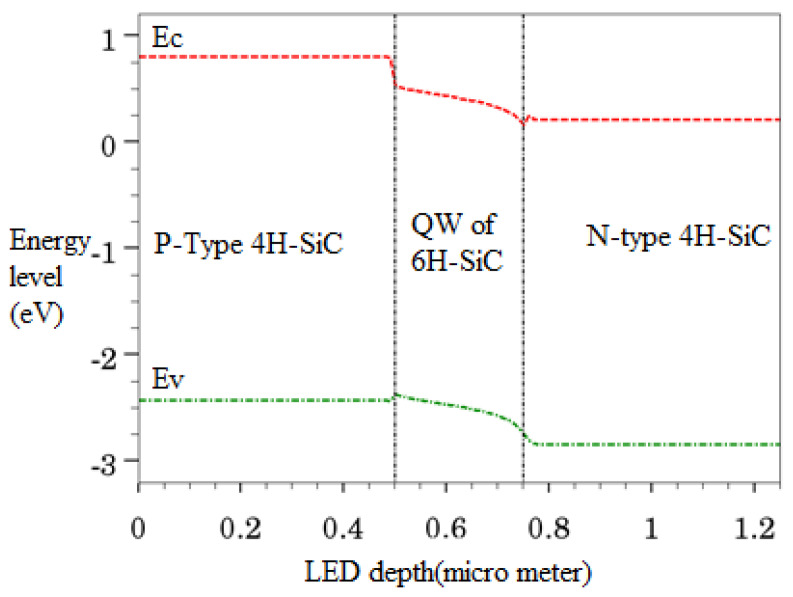
Energy band profile of SQW SiC LED as a function of its depth.

**Figure 6:**
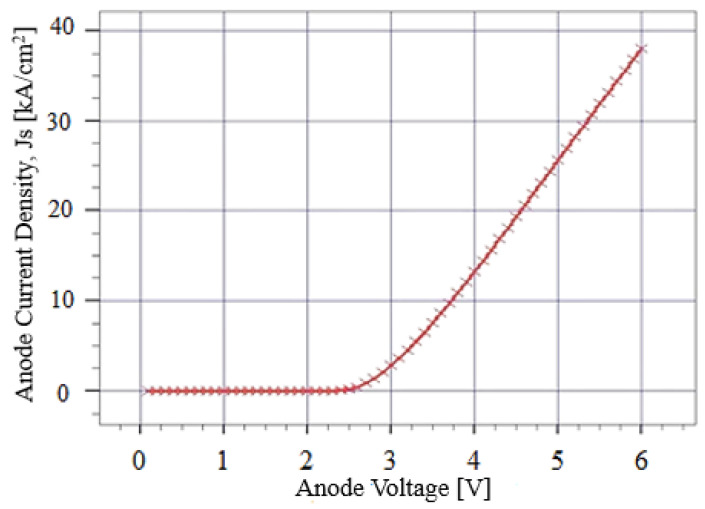
Anode voltage vs. anode current density of simulated SiC LED.

Energy band profile as a function of LED depth has been shown in Figure 5. The simulated device is a double hetero-structure, containing a quantum-well formed by intrinsic 6H-SiC between p-type 4H-SiC and n-type 4H-SiC. Band bending at each hetero-junction interface can be observed in Figure 5. The charge carrier can diffuse easily through the hetero-junction interface layers of the simulated device. Diffusion and tunneling are the key mechanisms that govern the transportation of charge carriers through heterostructure devices. The diffusion process controls the uniformity of current spreading, whereas the tunneling phenomenon contributes to limit the operational voltage of the device [49]. When an external voltage is applied at the electrodes of the device, the charge carriers spread widely due to the presence of the QW in the structure. A bias voltage is applied and current density has been measured for the simulated device as shown in Figure 6. The device starts to conduct a significant amount of current at approximately 2.6 V. After turning on, the current density starts to increase gradually and reaches to a maximum value of 39 kA/cm^2^ at approximately 6 V as shown in Figure 6. The formation of the QW in the simulated device indorses the vertical injection of the current and consequently reduces the turn-on voltage of the device.

### 3.2. Luminous Power of Simulated SiC LED

**Figure 7:**
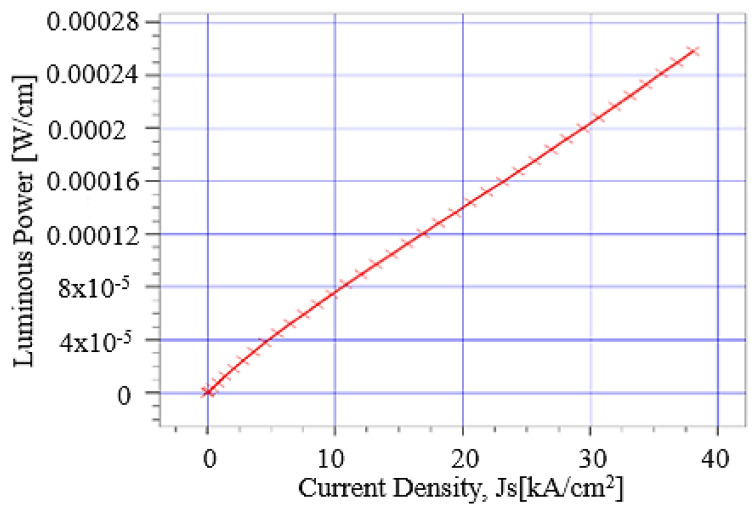
Anode current density vs. luminous power of simulated SiC LED.

A voltage bias of 0 to 6 V has been applied at the anode of the simulated LED. To analyze the luminous power output of the simulated LED, we need to know the current density (J_s_). Luminous power output as a function of current density has been given in Figure 7. At turn-on voltage of 2.6 V, the current density reaches the value of approximately 5 kA/cm^2^, and luminous power at this point is 4 × 10^−05^ W/cm, which is quite low. Current density of simulated LED gradually increases after turn-on voltage. At bias voltage of 6 V, the current density reached its maximum value, as shown in Figure 6. At this maximum value of 39 kA/cm^2^ current density, luminous power is 0.00028 W/cm. Although it is a SQW LED, still the luminous power is not so low. If we add multiple quantum wells (MQW) in this structure, this would definitely increase the luminous power output of the device. Because LED devices based on periodic MQW have high luminous power due to several reasons.

MQWs increase current spreading and charge carriers’ confinement. Ionization of electrons at high bias voltages increases the possibility of recombination of charge carriers [50]. However, we only introduced a SQW to reduce the complexity in fabrication and cost of the device.

### 3.3. Power Spectral Density of Simulated SiC LED

**Figure 8:**
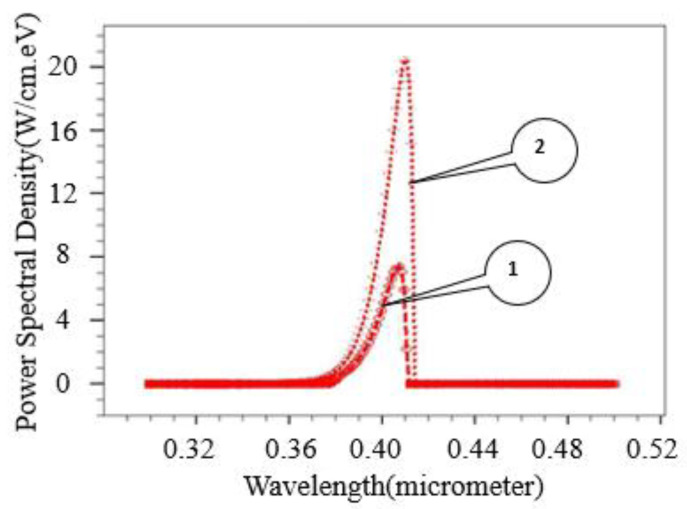
Wavelength vs. power spectral density of simulated SiC-based SQW LED. (Where curve-1 is at an anode voltage of 4 V and curve-2 is at an anode voltage of 6 V).

Power spectral density as a function of wavelength has been measured at two fixed voltages (4 V and 6 V) in TCAD. Emitted power spectral density of the simulated LED has been extracted using the TONYPLOT module of TCAD and shown in Figure 8. Our proposed device obtained a power output of 7 W/(cm·eV) at the wavelength of λ = 405 nm for fixed V_bias_ = 4 V, as shown in Figure 8 (curve-1). Whereas, the same device achieved a power output of 21 W/(cm·eV) at λ = 410 nm for V_bias_ = 6 V, as shown by curve-2 of Figure 8. For QW SiC-based LED height and width of spectral density would be higher compared to that of without QW. We could not find any similar edge LED structure in the literature for comparison that is purely based on SiC-SiC wafers considering the diffusion bonding approach.

### 3.4. Calculations for Luminous Efficiency and External Quantum Efficiency of Simulated SiC LED

The performance of LED is evaluated on the basis of percentage luminous efficiency and percentage external quantum efficiency (EQE). For these calculations, the simulated LED structure is fixed biased at V_bias_ = 6 V. After that, the radiative recombination rate and total recombination have been measured at the current density of 39 kA/cm^2^ using the TONYPLOT module. To calculate the percentage luminous efficiency of simulated LED, radiative recombination rate (r) has been divided with total recombination (T) and percentage luminous efficiency has been calculated, as given in Table 2. Our simulated structure showed 25% luminous efficiency. This efficiency is quite significant for a SQW LED. Furthermore, the percentage of external quantum efficiency of simulated LED has also been calculated. For these calculations, flux spectral density (Φ) of LED has been measured at the current density of 39 kA/cm^2^ using the TONYPLOT module. Then this flux spectral density is multiplied with charge q, where q = 1.602 × 10^−19^ Coulombs. Finally, this value is divided by bias current density to obtain external quantum efficiency. All these calculations and values have been given in Table 3. Our simulated LED showed 16.43% EQE. This efficiency is also quite good for SQW SiC LED. The formulae used for the calculation of luminous efficiency and quantum efficiency have been taken from the literature [50]. Both obtained efficiencies of our proposed LED can be optimized and customized numerically by choosing appropriate physical models and tuning of material parameters.

### 3.5. Comparison of Luminous Efficiency and External Quantum Efficiency of Simulated SiC LED with Literature

In this section, the luminous and external quantum efficiencies of simulated SiC LED have been compared with the similar LED structure reported in the literature. Epitaxial growth of SiC polytypes is extremely difficult and costly due to its ability to resist extremely high temperatures. Therefore, to avoid complications of the fabrication process and reduce the cost caused by the epitaxial growth techniques, a single QW has been used in our proposed LED structure. As we opt for the fabrication of SiC-based LED with a novel technique, called diffusion welding which is a cheap and simple fabrication technique. The comparison of luminous and external quantum efficiencies of our simulated SQW SiC LED has been done with the similar structure of GaN MQW LED reported in the literature. Despite the presence of SQW, our device exhibited impressive luminous and external quantum efficiencies compared to that of the MQW LED device reported in the literature [50]. The comparison of the results has been tabulated in Table 4 and Table 5. The reason for the high luminous and external quantum efficiencies of the GaN-based LED device reported in the literature is the presence of MQWs. (i.e., device A, B, and C have been taken from the literature [50]).

## 4. Conclusions

In this article, a novel design of SiC-based edge-emitting LED has been simulated by realizing a novel technique; diffusion welding/bonding. We proposed a unique combination of SiC-SiC polytype-based heterostructure LED by direct bonding of SiC wafers. This type of SiC-based LED has not yet been reported in the existing literature since it is extremely challenging to join SiC wafers directly due to their ability to resist even extremely elevated temperatures. Considering the research gap, the comparative performance analysis of the devised LED structure has been carried out with GaN-based LED reported in the literature. However, it is noteworthy that comparative LED possesses the attributes of MQW, whereas our device has been realized with SQW. Moreover, we delineated a novel direct bonding technique for joining SiC-SiC wafers to reduce the complexity and fabrication cost. Our simulated LED device exhibited promising results in terms of luminous efficiency and external quantum efficiency. The simulated device achieved 25% luminous efficiency and 16.43% external quantum efficiency with only one quantum-well formed by the active layer of 6H-SiC. Prospective fabrication of our proposed device with diffusion welding will dramatically reduce the device cost since diffusion welding techniques has several economic advantages over state-of-the-art epitaxial techniques. This process is very simple and allows high material utilization. It reduces the fabrication complexity and processing time. The characteristics of our proposed LED device can be customized by choosing appropriate materials with varying bandgaps to obtain the wavelength of emitted light in the desired wavelength range. Catalytic etching has been proposed for polishing of 4H-SiC wafers to get a flatter surface at the atomic level in order to improve the bonding capability of SiC wafers for the prospective physical fabrication of the device.

## Figures and Tables

**Table 1:** Materials parameters used in SILVACO TCAD for simulated SiC LED [47].

Symbol	Quantity	Value
E_g1_	Bandgap of 4H-SiC	3.24 eV
E_g2_	Bandgap of 6H-SiC	3.00 eV
Permittivity	6H-SiC	9.66
Permittivity	4H-SiC	9.7
Affinity	6H-SiC	3.00 eV
Affinity	4H-SiC	3.24 eV
*N*_c_	Electron density of states for 6H-SiC	7.68 × 10^18^ cm^−3^
*N*_c_	Electron density of states for 4H-SiC	1.66 × 10^19^ cm^−3^
*N*_v_	Holes density of states for 6H-SiC	4.76 × 10^+18^
*N*_v_	Holes density of states for 4H-SiC(per cc)	3.30 × 10^+19^
el	Life time of electrons for 6H-SiC	1.00 × 10^−7^
el	Life time of electrons for 4H-SiC	1.00 × 10^−7^
ho	Life time of holes for 4H-SiC	1.00 × 10^−7^
ho	Life time of holes for 6H-SiC	1.00 × 10^−7^
MUNO	Mobility of electrons for 6H-SiC	330 cm^2^/(V·s)
MUNO	Mobility of electrons for 4H-SiC	440 cm^2^/(V·s)
MUPO	Mobility of holes for 6H-SiC	300 cm^2^/(V·s)
MUPO	Mobility of holes for 4H-SiC	124 cm^2^/(V·s)

**Table 2:** Luminous efficiency calculations for simulated SiC LED at 39 kA/cm^2^ bias.

Device	Radiative Rate cm^3^/s (r)	Recombination Rate cm^3^/s (T)	%Luminuous Efficiency (ƞ = r/T × 100)
SQW SiC LED	5 × 10^14^	2 × 10^15^	25

**Table 3:** External quantum efficiency calculation for simulated LED at 6 V.

Device	Flux Spectral Density [s cm eV]^−1^ (Φ)	Bias Current kA/cm^2^ (Js)	%External Quantum Efficiency (%EQE = (q Φ/Js) × 100)
SQW SiC LED	4 × 10^19^	39	16.43

**Table 4:** Comparison of the luminous efficiencies of simulated. SiC LED with the literature [50].

Device	Bias Current kA/cm^2^ (Js)	%Luminous Efficiency (ƞ = r/T × 100)
SQW SiC LED	39	25
Device A *	5.62	79.8
Device B *	5.62	82.5
Device C *	5.62	81.0

* These LED structures have multiple quantum wells.

**Table 5:** Comparison of the external quantum efficiencies of simulated. SiC LED with literature [50].

Device	Bias Current kA/cm^2^ (Js)	%External Quantum Efficiency (%EQE = (q Φ/Js) × 100)
SQW SiC LED	39	16.43
Device A *	5.62	19.3
Device B *	12.32	25.4
Device C *	12.02	24.0

* These LED structures have multiple quantum wells.
